# Whole-body MRI evaluation in neurofibromatosis type 1 patients younger than 3 years old and the genetic contribution to disease progression

**DOI:** 10.1186/s13023-022-02174-3

**Published:** 2022-01-29

**Authors:** Eungu Kang, Yoon-Myung Kim, Yunha Choi, Yena Lee, JunYoung Kim, In Hee Choi, Han-Wook Yoo, Hee Mang Yoon, Beom Hee Lee

**Affiliations:** 1 Department of Pediatrics, Korea University Ansan Hospital, Korea University College of Medicine, Ansan, Korea; 2 Department of Pediatrics, Gangneung Asan Hospital, Gangneung, Korea; 3 Department of Pediatrics, Asan Medical Center Children’s Hospital, University of Ulsan College of Medicine, 88, Olympic-ro 43-Gil, Songpa-Gu, Seoul, 05505 Korea; 4 Department of Genetic Counseling, University of Ulsan College of Medicine, Seoul, Korea; 5 Medical Genetics, Asan Medical Center, University of Ulsan College of Medicine, Seoul, Korea; 6 Department of Radiology and Research Institute of Radiology, Asan Medical Center, University of Ulsan College of Medicine, 88, Olympic-ro 43-Gil, Songpa-Gu, Seoul, 05505 Korea

**Keywords:** Neurofibromatosis 1, NF1, Magnetic resonance imaging, Genotype–phenotype correlation

## Abstract

**Background:**

Neurofibromatosis type 1 (NF1) is a common human genetic disease with age-dependent phenotype progression. The overview of clinical and radiological findings evaluated by whole-body magnetic resonance imaging (WBMRI) in NF1 patients < 3 years old assessed with a genetic contribution to disease progression is presented herein.

**Methods:**

This study included 70 clinically or genetically diagnosed NF1 patients who received WBMRI before 3 years old. Clinical, genetic, and radiologic features were collected by retrospective chart review. In NF1^+^, widely spread diffuse cutaneous neurofibromas, developmental delay, autism, seizure, cardiac abnormalities, hearing defect, optic pathway glioma, severe plexiform neurofibromas (> 3 cm in diameter, disfigurement, accompanying pain, bony destruction, or located para-aortic area), brain tumors, nerve root tumors, malignant peripheral nerve sheath tumors, moyamoya disease, and bony dysplasia were included.

**Results:**

The age at WBMRI was 1.6 ± 0.7 years old, and *NF1* mutations were found in 66 patients (94.3%). Focal areas of signal intensity (FASI) were the most common WBMRI finding (66.1%), followed by optic pathway glioma (15.7%), spine dural ectasia (12.9%), and plexiform neurofibromas (10.0%). Plexiform neurofibromas and NF1^+^ were more prevalent in familial case (28.7% vs 5.7%, *p* = 0.030; 71.4% vs 30.2%, *p* = 0.011). Follow-up WBMRI was conducted in 42 patients (23 girls and 19 boys) after 1.21 ± 0.50 years. FASI and radiologic progression were more frequent in patients with mutations involving GTPase activating protein-related domain (77.8% vs 52.4%, *p* = 0.047; 46.2% vs 7.7%, *p* = 0.029).

**Conclusions:**

WBMRI provides important information for the clinical care for young pediatric NF1 patients. As NF1 progresses in even these young patients, and is related to family history and the affected *NF1* domains, serial evaluation with WBMRI should be assessed based on the clinical and genetic features for the patients’ best care.

**Supplementary Information:**

The online version contains supplementary material available at 10.1186/s13023-022-02174-3.

## Introduction

Neurofibromatosis type 1 (NF1; OMIM#16220) is one of the most common genetic diseases with an incidence of approximately one in 3000 individuals [1, 2]. It is characterized by heterogeneous involvements of multiple organ systems with variable expressivity [3]. NF1 is caused by loss of function mutations in the *NF1* gene, which is located at the 17q11.2 and encodes neurofibromin [4]. Neurofibromin is a multifunctional protein that is essential for embryonic development and is ubiquitously expressed. However, the highest levels are found in the neuronal cells [5]. As a main regulator of the rat sarcoma virus (RAS)–mitogen-activated protein kinases (MAPK) pathway, it affects various cellular processes (e.g., proliferation, growth, division, survival, and migration) in cells of different tissues [6, 7]. Neurofibromin consists of multiple domains: an N-terminal cysteine-serine rich domain (CSRD), a central GTPase-activating protein-related domain (GRD) with a tubulin-binding domain in N-terminus, a phospholipid- and protein-interaction domain, and a C-terminal domain [8].

NF1 is a tumor-predisposing disease with a higher susceptibility to several types of tumors (e.g., neurofibromas, peripheral nerve sheath tumors, central nervous system gliomas, pheochromocytoma, juvenile myelomonocytic leukemia, and rhabdomyosarcoma) [9, 10]. In NF1 patients, the screening, early diagnosis, and long-term follow-up of various tumor types are important. Considering that tumors are distributed widely across the anatomic regions in NF1 patients, whole-body magnetic resonance imaging (WBMRI) can be suggested as the most suitable imaging modality for NF1 patients. Moreover, WBMRI is a practical approach not only for tumor detection but also for tumor burden evaluation, tumor characterization, and treatment response assessment [11].

The NF1 diagnosis is established according to the National Institutes of Health (NIH) Consensus Development Conference diagnostic criteria in individuals with more than two typical clinical manifestations and/or family history [12, 13]. Typical NF1 clinical features include multiple café-au lait macules (CALMs), axillary freckling, cutaneous neurofibromas, plexiform neurofibromas (PN), Lisch nodules, optic pathway gliomas, and skeletal deformities [14–16]. Vascular anomalies, endocrine disorders, and neurodevelopmental disorders are also related. The NF1 clinical features are an age-dependent process and continuously evolve with age.

This study focused on the clinical and radiological findings of NF1 in early childhood which is scarce. The overview of clinical and radiological findings evaluated by WBMRI in NF1 patients younger than 3 years old with the assessment of genetic contribution to disease progression is presented herein.

## Methods

### Patients

This study included 70 consecutive Korean patients < 3 years old who are clinically or genetically diagnosed with NF1 between February 2017 and April 2020 at the Department of Medical Genetics, Asan Medical Center Children’s Hospital, Seoul, Korea. All patients underwent WBMRI < 3 years old. This study was approved by the Institutional Review Board at the Asan Medical Center (IRB number 2021-1084), and appropriate written informed consent was obtained from the patient’s parents for *NF1* gene testing.

### Clinical data

Patient data on gender, age, inheritance mode, clinical and radiological findings, and molecular analysis were collected using retrospective chart review.

The NF1 diagnosis was established in patients who meet the previous NIH diagnostic criteria and/or harbor the heterozygous pathogenic variants in *NF1* [12, 13]. A previous report [17] subgrouped the phenotypes that require medical attention as NF1-plus (NF1^+^), which includes widely spread diffuse cutaneous neurofibromas, developmental delay, autism, seizure, cardiac abnormalities, hearing defect, optic pathway glioma, severe PN (> 3 cm in diameter, disfigurement, accompanying pain, bony destruction, or located para-aortic area), brain tumors, nerve root tumors, malignant peripheral nerve sheath tumors, moyamoya disease, or bony dysplasia [17]. Clinical and radiological progression was defined as the development of the new items included in NF1^+^, their progression, or increased PN size.

Short stature was defined as a height below 2.0 standard deviation score for age and gender compared with the Korean population-based reference [18]. Development was evaluated with the Korean infant and child development test (KICDT) [19], which was developed by the Development Education Enacting Subcommittee of the Korean Pediatrics Academy. KICDT was designed to assess development in five functional domains: gross motor, fine motor, social–personal, language, and cognitive–adaptive skills. The developmental quotient (DQ = (developmental age/chronological age) × 100) lower than 80 was regarded as abnormal development.

### Whole-body MRI and imaging analysis

3T MR imager (Ingenia, Philips Medical Systems, Best, The Netherlands) with an integrated body coil was used to acquire WBMRI in each patient. Each patient was examined from head to toe in the supine position. In the institution of this study, WBMRI for patients with NF1 includes the entire body (using a coronal and sagittal short tau inversion recovery image) and brain (using axial T1- and T2-weighted images and fluid-attenuated inversion recovery image) imaging. Additionally, coronal T2-weighted images with fat suppression from bilateral orbits through optic chiasm were obtained to evaluate optic pathway abnormality. All patients were sedated during examination according to the institutional protocol by a pediatric anesthesiologist. Intravenous contrast material was not administered. Detailed imaging parameters are summarized in Additional file 1: Table S1. Moreover, WBMRI was retrospectively reviewed by a pediatric radiologist (HMY with over 8 years of clinical experience in pediatric radiology) who was blinded to the genetic and clinical information.

### Molecular analysis

The genomic DNA was isolated from peripheral blood leukocytes using a Gentra Puregene Blood kit (Qiagen, Hilden, Germany). Sanger sequencing was performed for all coding 57 exons and exon–intron boundaries of NF1 (NM_000267.3). The nested polymerase chain reaction (PCR) was done in each of the five long PCR products (6–13 kb) which was amplified using NF-1 specific primers to avoid homologous domain amplification as previously described [17]. Multiplex ligation-dependent probe amplification analyses were performed using SALSA MLPA Probemix P081 and P082 (MRC Holland, Amsterdam, The Netherlands).

**Fig. 1:**
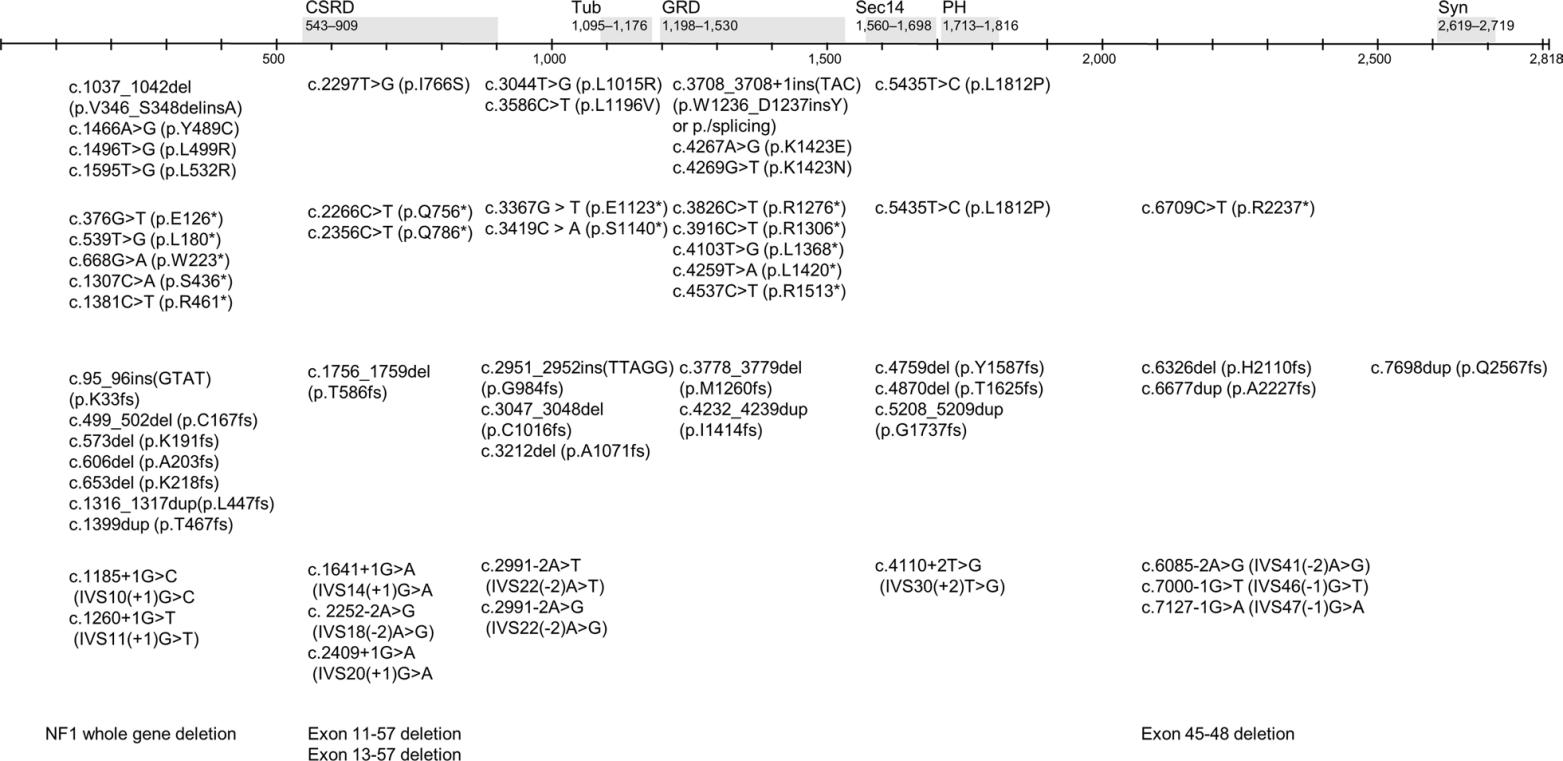
The locations of NF1 functional domains and the distribution of the *NF1* mutations. The numbering of the domain boundaries is shown in amino acids. *CSRD* N-terminal cysteine-serine rich domain, *Tub* tubulin-binding domain, *GRD GAP* GTPase-activating protein-related domain, *Sec14* Sec14 homology-like domain, *PH* pleckstrin homology-like domain, *Syn* syndecan

The NF1 mutations were classified according to the location of the variants because NF1 protein domains are related to different biochemical functions (Fig. 1) [20]. Nonsense, frameshift, or splicing mutations were considered as all subsequent domains were affected.

### Statistical analysis

Two-tailed Fisher’s exact test was performed for comparison of categorical variables. A *P* value < 0.05 was considered statistically significant. All statistical analyses were performed with SPSS version 21 for Windows (SPSS Inc., Chicago, IL, USA).

## Results

### Clinical characteristics and whole-body MRI findings of the patients

WBMRI was performed on 70 children with NF1 (41 girls and 29 boys) at the mean age of 1.6 ± 0.7 years. The cohort included 14 familial cases, 53 sporadic cases and 3 patients with unknown inheritance patients. The clinical and radiologic characteristics of these patients are shown in Table 1.

**Table 1:** Baseline clinical and radiological findings of NF1 patients younger than 3 years old

	0–1 year (*N* = 8)	1–2 years (*N* = 45)	2–3 years (*N* = 17)	*p* value
Gender				
Male	3/8 (37.5%)	15/45 (33.3%)	11/17 (64.7%)	0.087
Inheritance				
Familial	0 (0.0%)	7/45(15.6%)	7/17 (41.2%)	0.011
Sporadic	6/8 (75.0%)	37/45 (82.2%)	10/17 (58.8%)	
Unknown	2/8 (25.0%)	1/45 (2.2%)	0/17 (0.0%)	
Clinical findings				
Café-au lait macules	8/8 (100%)	45/45 (100%)	17/17 (100%)	–
Axillary freckling	2/5 (40.0%)	14/42 (33.3%)	12/15 (80.0%)	0.005
Cutaneous neurofibromas	1/5 (20.0%)	4/23 (17.4%)	2/12 (16.7%)	1.000
Relative macrocephaly	2/8 (25.0%)	22/42 (52.4%)	13/17 (76.5%)	0.052
Lisch nodules	0/2 (0.0%)	3/10 (30.0%)	5/13 (38.5%)	0.205
Short stature	0/8 (0.0%)	1/45 (2.2%)	1/17 (5.9%)	0.590
Developmental delay	0/4 (0.0%)	2/26 (7.7%)	2/12 (16.7%)	0.721
Seizure	1/3 (33.3%)	1/26 (3.8%)	0/9 (0.0%)	0.205
Cardiac anomaly	1/2 (50.0%)	1/8 (12.5%)	–	0.378
Hearing defect	–	0/12 (0.0%)	0/6 (0.0%)	–
Radiologic findings				
FASI	6/8 (75.0%)	26/45 (57.8%)	15/17 (88.2%)	0.056
Optic pathway glioma	0/8 (0.0%)	4/45 (8.9%)	7/17 (41.2%)	0.006
Moyamoya disease	0/8 (0.0%)	1/45 (2.2%)	0/17 (0.0%)	1.000
Aneurysm	0/8(0.0%)	0/45 (0.0%)	0/17 (0.0%)	–
Sphenoid wing dysplasia	0/8 (0.0%)	3/45 (6.7%)	2/17 (11.8%)	0.791
Superficial neurofibromas	0/8 (0.0%)	2/45 (4.4%)	0/17 (0.0%)	1.000
Deep localized neurofibromas	0/8 (0.0%)	2/45 (4.4%)	2/17 (11.8%)	0.574
Plexiform neurofibromas	1/8 (12.5%)	3/45 (6.7%)	4/17 (23.5%)	0.171
Scoliosis	0/8 (0.0%)	0/45 (0.0%)	1/17 (5.9%)	0.357
Spinal dysplasia	0/8 (0.0%)	0/45 (0.0%)	0/17 (0.0%)	–
Spinal dural ectasia	0/8 (0.0%)	6/45 (13.3%)	3/17 (24.3%)	0.572
Long bond dysplasia	0/8 (0.0%)	4/45 (8.9%)	2/17 (11.8%)	0.842
MPNST	0/8 (0.0%)	0/45 (0.0%)	0/17 (0.0%)	–
NF1^+^	2/8 (25.0%)	13/45 (28.9%)	12/17 (70.6%)	0.008

*FASI* focal areas of signal intensity, *MPNST* malignant peripheral nerve sheath tumor

**Fig. 2:**
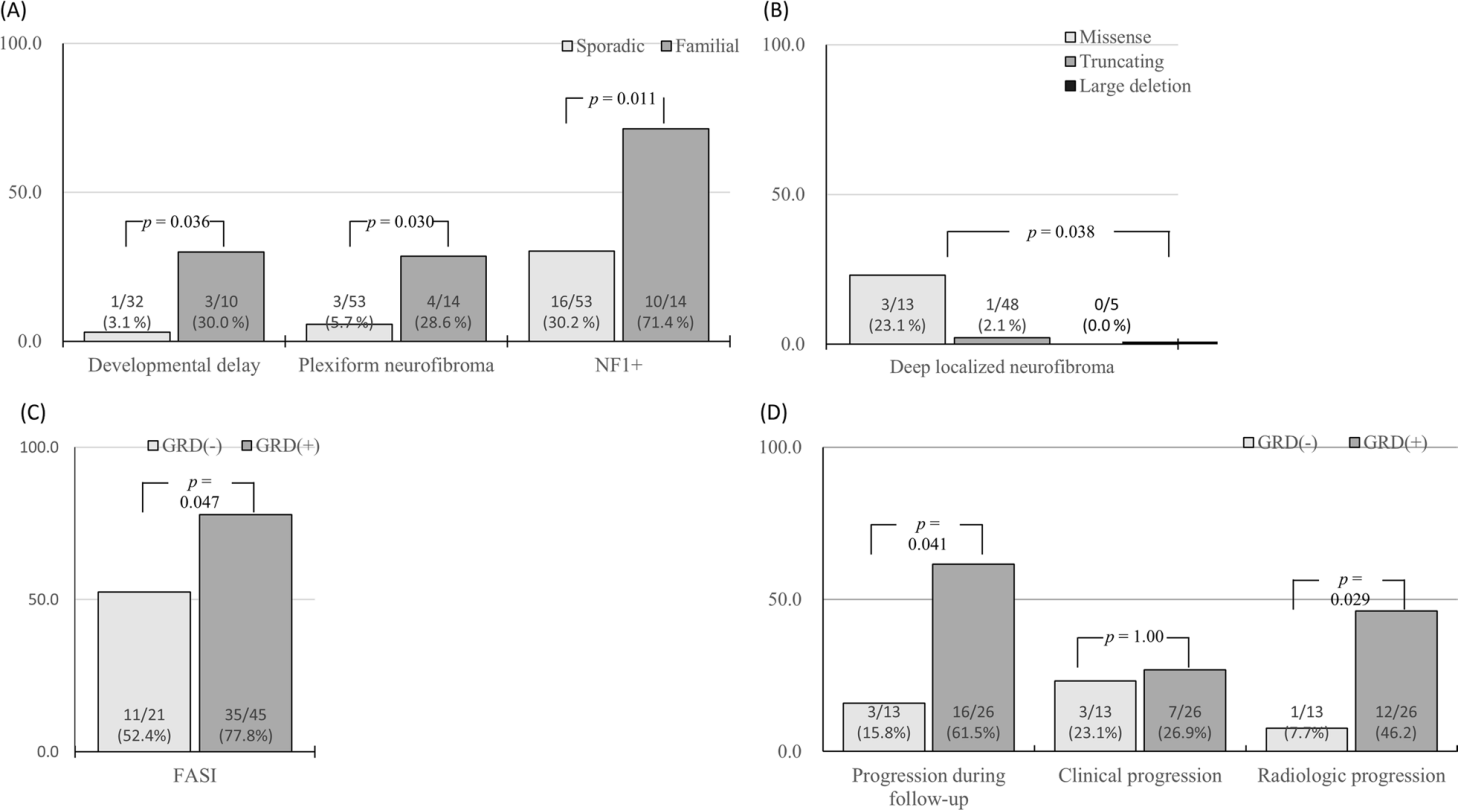
The frequency of clinical and radiologic manifestations according to inheritance (**A**), mutation type (**B**), and affected domains by NF1 mutations (**C**, **D**)

The most frequent NF1-related typical manifestation was CALMs (70/70, 100%), followed by relative macrocephaly (37/67, 55.2%), axillary freckling (28/62, 45.2%), Lisch nodules (8/25, 32.0%), and cutaneous neurofibromas (7/40, 17.5%). Neurologic problems, such as developmental delay or seizure, were reported in 9.5% (4/42) and 5.3% (2/38) of patients, respectively. Short stature was reported in 2.9% (2/70) of patients. Atrial septal defect was reported in one patient, which was spontaneously closed during follow-up. Developmental delay (30.0% vs 3.1%, *p* = 0.036), PN (28.6% vs 5.7%, *p* = 0.030), and NF1^+^ (71.4% vs 30.2%, *p* = 0.011) was more commonly observed in familial cases (Fig. 2A).

**Fig. 3:**
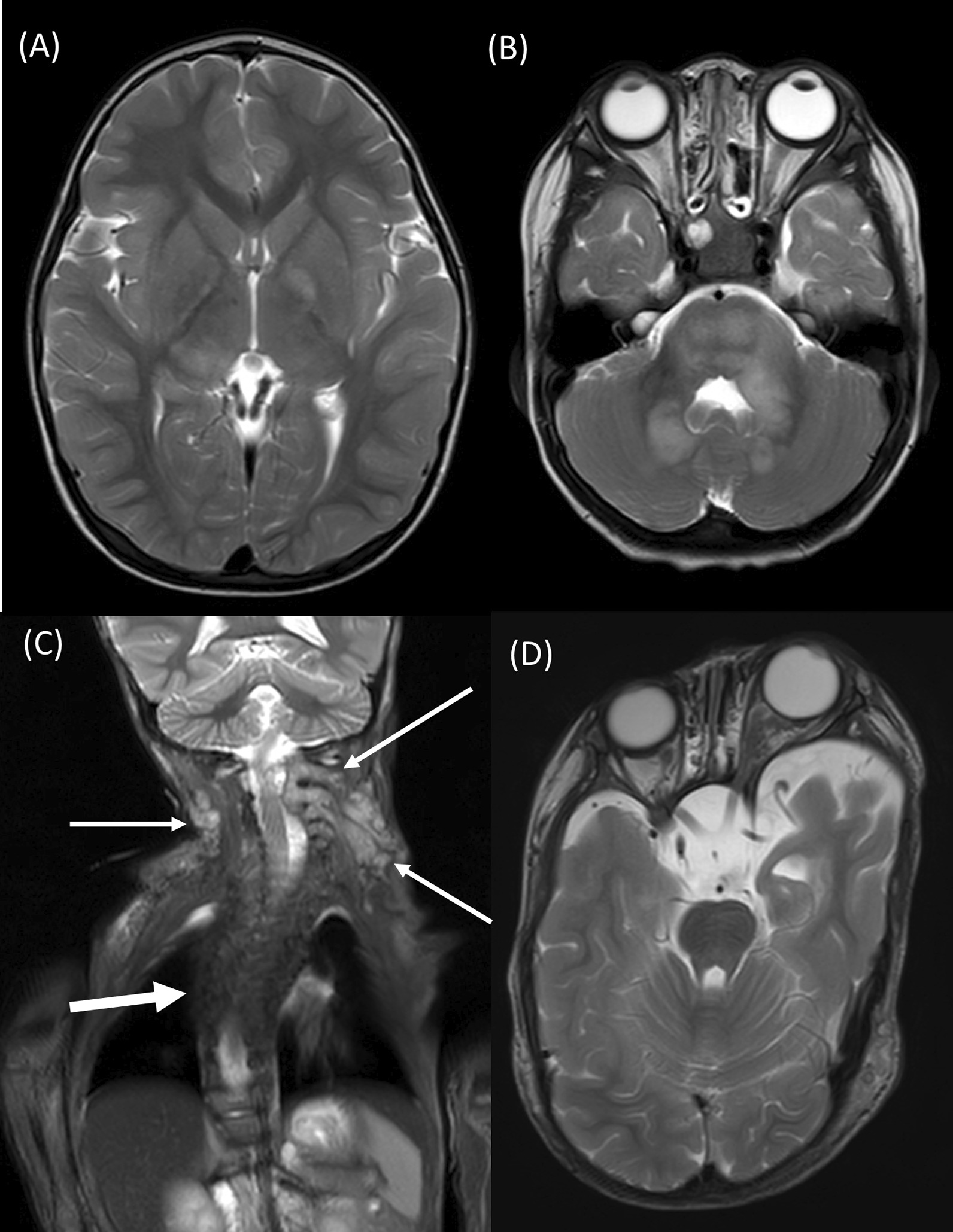
**A**, **B** A 2.67-year-old boy with genetically confirmed NF1 (*NF1* c.3212del (p.A1071fs)). T2 hyperintensity and focal areas of signal intensity (FASI) were noted in the left globus pallidus, right thalamus, pons, left middle cerebellar peduncle, and bilateral cerebellar white matter (arrows). **C**, **D** A 2.75-years-old male with genetically confirmed NF1 (*NF1* c.95_96insGTAT (p.K33fs)). **C** Image from whole-body coronal short tau inversion recovery showed plexiform neurofibromas at the bilateral cervical spinal nerve roots with mass effect to the dural sac (arrows). Thoracic scoliosis with right-sided convexity (thick arrow) was also noted. **D** Axial T2WI of the brain showed bilateral sphenoid dysplasia (more severe in the left side) with anterior bulging of the bilateral temporal lobe and left proptosis

The most frequent WBMRI finding at the first evaluation was focal areas of signal intensity (FASI; 47/70, 67.1%), followed by optic pathway glioma (11/70, 15.7%), bony dysplasia (10/70, 14.3%), suspicious mild spinal dura ectasia (9/70, 12.9%), and PN (8/70, 11.4%). Regarding FASI, the cerebellum (72.3%) was the most frequently involved site, followed by basal ganglia (38.3%), brainstem (31.9%), internal capsule (23.4%), and thalamus (10.6%). Less frequently, FASI was also found in the left parietal cortex (1/47, 2.1%), dentate (1/47, 2.1%), midbrain (1/47, 2.1%), centrum semiovale (1/47, 2.1%), and anterior commissure (1/47, 2.1%). The involved bony dysplasia sites were sphenoid wing (5/10, 50.0%), tibia and/or fibula (4/10, 40.0%), pubis and ischium (1/10, 10.0%), and sacrum (1/10, 10.0%). PN located on the superficial areas in 4 patients (right parotid, superficial neck, left cheek, and right arm/left periauricular area) were visible and palpable on physical examination. Representative cases are shown in Fig. 3.

**Fig. 4:**
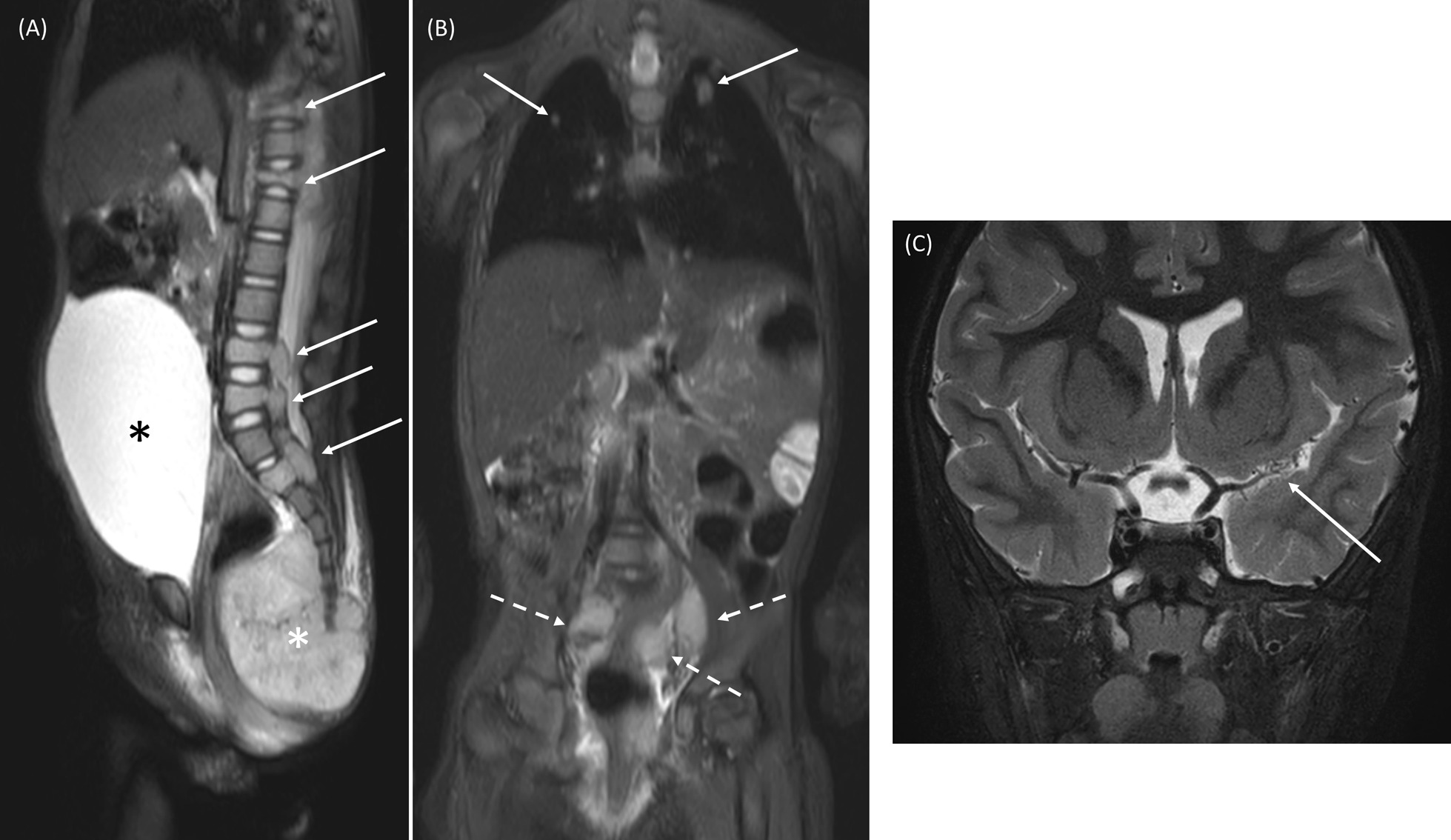
**A**, **B** A 1.41-year-old girl with genetically confirmed NF1 (*NF1* c.4110 + 2T > G (IVS30(+ 2)T > G)). **A** Sagittal short tau inversion recovery (STIR) shows a large sacrococcygeal mass (white asterisk) which was a malignant germ cell tumor (yolk sac tumor) and multiple metastatic bone lesions (arrows). The urinary bladder (black asterisk) is markedly distended probably due to bladder outlet obstruction caused by the mass. **B** On coronal STIR images, lung (arrows) and lymph nodes metastasis (broken arrows) were noted. **C** A 1.75-year-old boy with NF1 (*NF1* c.1307C > A (p.S436*)) was diagnosed with West syndrome. Coronal T2WI with fat suppression depicts segmental luminal narrowing of the left middle cerebral artery with multiple basal collaterals (arrows). A horseshoe kidney (not shown) was also noted on WBMRI

Importantly, a few cases with extraordinary manifestations were noted. A 1.5-year-old girl (*NF1* c.4110 + 2T > G (IVS30(+ 2)T > G)) had a germ cell tumor in the pelvic area with bone, lung, and lymph node metastasis (Fig. 4A, B). A 1.8-year-old boy (*NF1* c.1307C > A (p.S436*)) with infantile spasm, hypsarrythmia, and developmental arrest was diagnosed with West syndrome and had moyamoya disease and a horseshoe kidney in WBMRI (Fig. 4C). A 1.1-year-old girl (*NF1* c.6326del (p.H2110fs)) was removed cervical nerve root tumor causing the spinal cord compression with the neurologic deficit, which was detected by WBMRI examined at the age of 11 months.

The remaining 11 patients (15.7%, four girls and seven boys; mean age, 1.4 ± 0.4 years) had no radiologic abnormalities related to NF1 on WBMRI. No significant differences in gender (22 males and 37 females with WBMRI abnormality *vs* seven males and four females without WBMRI abnormality, *p* = 0.181) or age (1.6 ± 0.7 years vs 1.4 ± 0.4 years, *p* = 0.07) were noted between the patients with and without abnormalities in WBMRI.

When the frequency of the clinical phenotypes was analyzed according to age at WBMRI evaluation, the frequency of most phenotypes increased in patients 2–3 years old. Moreover, the frequency of axillary freckling, optic pathway glioma, and NF1^+^ significantly increased (*P* < 0.05; Table 1).

### NF1 diagnosis

Fifty-two (74.3%) patients fulfilled the previous NIH diagnostic criteria. Among 18 patients who had CALMs in NIH diagnostic criteria and confirmed NF1 by genetic testing, 50% (4/8) of the patients were younger than 1 year old, and 31.1% (14/45) of the patients were 1–2 years old. In patients who did not meet the previous NIH criteria, relative macrocephaly, FASI, sphenoid dura ectasia, spinal dura ectasia, and seizure were observed in nine (50.0%), eight (44.4%), six (33.3%), three (33.3%), and one (5.6%) patient, respectively.

In contrast, all patients met the revised diagnostic criteria for NF1; 66 patients with at least CALMs and heterozygous pathogenic *NF1* variants; 3 patients with CALMs and axillary freckling; 1 patient with CALMs and a positive family history.

### Molecular findings and genotype–phenotype correlation

Long-range PCR and sequencing of gDNA and MLPA analyses identified *NF1* mutations in 87.1% and 7.1% of patients, respectively. The mutation type distributions were frameshift mutations (*N* = 21, 31.8%), nonsense mutations (*N* = 16, 24.2%), missense mutations (*N* = 13, 19.7%), splice-site mutations (*N* = 11, 16.7%), and large deletions (*N* = 5, 7.6%; Fig. 1). Deep localized neurofibroma was more prevalent in missense mutations than other mutation types (Fig. 2B).

The NF1 functional domains affected by each NF1 mutation were assessed. Syn domain was most commonly affected (52/66, 78.8%), followed by pleckstrin homology (PH; 46/66, 69.7%), GRD (45/66, 68.2%), Sec14 homology-like domain (SEC14; 44/66, 67.5%), tubulin binding (35/66, 53.0%), and CSRD (26/66, 39.4%) domains. The frequency of phenotypes were assessed according to the affected domains, and FASI was more prevalent (77.8% vs 52.4%, *p* = 0.047; Fig. 2C, Additional file 1: Table S2) in patients with NF1 mutation affecting GRD involvement.

### Progression during follow-up

Follow-up MRI was performed in 42 (60.0%) patients (23 girls and 19 boys; mean age, 2.78 ± 0.91 years). The mean duration between the baseline and follow-up MRIs was 1.21 ± 0.50 years. In addition, 19 (45.2%) patients exhibited progression during follow-up: clinical, radiological, and both clinical and radiological progressions were shown in six, nine, and four patients, respectively. The detailed clinical and radiological findings of the patients who showed progression during follow-up are summarized in Additional file 1: Table S3 and a representative case was shown in Additional file 2: Fig. S1. The common phenotypes exhibiting progression were developmental delay in language (*N* = 7) or fine motor (*N* = 3) according to KICDT. The patient with West syndrome did not achieve any developmental milestones during follow-up. In the follow-up WBMRI, the radiologic aspects, optic nerve signal change or thickening, were newly developed in eight patients. Additionally, optic nerve signal change and severe PN (one patient), severe PN and diffuse superficial neurofibroma (one patient), nerve root tumor (one patient), brain tumor on pons (one patient), and neuroblastoma in the left adrenal gland (one patient) were also newly developed.

Clinical and genotypic features were compared between patients experiencing or not experiencing disease progression. Gender, inheritance mode, and mutations type were not associated with disease progression. However, disease progression was observed in higher proportion in patients with NF1 mutation affecting GRD (61.5% vs 15.8%, *p* = 0.041), SEC (64.0% vs 21.4%, *p* = 0.019), and PH (64.0% vs 21.4%, *p* = 0.019) domains. Radiological progression was especially more frequently observed in patients with *NF1* mutations affecting the GRD domain (46.2% vs. 7.7%, *p* = 0.029; Fig. 2D), whereas no significant differences were observed among those with NF1 mutations affecting other domains. No significant differences were noted in the prevalence of clinical progression among the affected domains of *NF1* mutations.

## Discussion

The current study investigates the clinical characteristics and WBMRI findings of NF1 patients younger than 3 years old and their genetic contribution to clinical severity and progression. Its diagnosis is suggested mainly based on clinical suspicion because typical NF1 features evolve with the age-related process, and only half of the patients fulfilled the previous NIH diagnostic criteria by 1 year old. Indeed, the diagnosis would not have been confirmed in about 25% of the patients in the current study if genetic testing was not done. Whereas, the revised diagnostic criteria, which included the pathogenic variants of NF1 as a new item, confirmed the diagnosis of NF1 in all patients under 3 years of age and led to the early recognition. A risk exists where some pediatric NF1 patients may remain undiagnosed and lose critical time for therapeutic intervention to prevent severe, irreversible complications. Thus, the detailed clinical, radiological, and genetic evaluation of these young NF1 patients (< 3 years old) provides some new and important insights into the general but precise care of NF1 in pediatric patients.

The current study expected, even in these young ages, that the clinical features including axillary freckling, optic pathway gliomas, and NF1^+^ evolved as age increases. Importantly, these features became more obvious in patients with a positive family history. Delayed development, PN, and skeletal abnormalities (e.g., sphenoid wing, long bone dysplasia, and scoliosis) also became more prevalent, although statistical significance was not achieved due to the small number of patients.

In NF1 patients, learning disability or behavior problems are relatively common and vary from 30 to 80% in frequency [21–23]. Yet, flank intellectual disability is uncommon. Its prevalence in the current study was relatively low because the initial evaluation was done by parents’ reports. However, KICDT revealed clinical progression related to developmental delay. In addition, most patients (9/10) showed a mild delay in language or fine motor domain with 70–75 of DQ, but severe retardation was noted in one patient with West syndrome.

Optic pathway gliomas are the most important central nervous system-associated tumors in NF1 patients younger than 6 years old (median age of presentation, 4.2 years old) [24]. Histologically, they are slow-growing benign tumors with a low risk of malignancy. However, they become symptomatic due to the space-occupying location [25, 26]. They can cause a rapid onset of proptosis with decreased visual acuity and lead to precocious puberty when located on the optic chiasm [26]. An ophthalmologic examination should be performed at least annually until 13 years old. However, routine screening and surveillance of the optic nerve and pathways by MRI in young NF1 patients without symptoms is controversial and not recommended [27, 28]. Detailed ophthalmologic examination is not easy in these young patients who seldom complain of visual symptoms. The current study found optic pathway glioma in 15.7% of patients and with some patients experiencing new development of optic pathway glioma during follow-up evaluation of WBMRI. With these regards, it should be seriously considered that the serial (maybe annual) WBMRI including optic pathway visualization may be necessary for young pediatric patients even though they do not show overt ophthalmological manifestations.

Various types of neurofibromas are classic NF1 manifestations. Cutaneous neurofibromas typically appear in adolescence, while PN often is detected early in childhood and is supposed to be congenital lesions [27, 29]. PN arises from one or multiple nerve trunks or branches that may be asymptomatic or can also cause significant morbidity including pain, adjacent structure compressions, and malignant transformation risks [30, 31]. Surgical PN excision is usually challenging due to interdigitating of tumors on adjacent structures and peripheral nerves as well as the extensive vascularity that can result in life-threatening hemorrhage [32]. The current observation suggests that about one-third of NF1 patients already have PN by 3 years old and progressive growth can be observed. Recently, there have been advancements in the medical therapy of various NF1 tumors including PN. Selumetinib, an oral selective MEK inhibitor, decreases PN and improves PN-related complications (e.g., pain, a limitation of physical activity and quality of life) [33]. Furthermore, serious complications associated with NF1 (e.g., germ cell tumor, brain stem glioma, and adrenal gland neuroblastoma) were also observed. Thus, WBMRI is not only an efficient method of detecting brain, optic nerve, and vascular abnormalities as well as internal tumors at initial and follow-up evaluation, but it also allows an accurate tumor response assessment to a new medical therapy without ionizing radiation exposure [11, 34]. Meanwhile, there are concerns about the risk of sedation and/or anesthesia in young children and further studies are required to assess the risks and the benefits of WBMRI in young NF1 children.

Most NF1 patients are diagnosed in childhood and even in infancy. Thus, parents are worried about the prognosis of their child. Predicting the natural course of each patient is difficult because NF1 is a lifelong evolving disease and a highly penetrant with variable expressivity. Due to the high spontaneous mutation rates across the *NF1* gene, more than 3000 different germline mutations have been identified with the extreme diversity of the *NF1* mutational spectrum [8, 35].

Identification of overall genotype–phenotype correlation may help provide information that can predict the prognosis. In the current study, missense mutations were associated with deep localizing tumors at initial evaluation. Several specific genotype–phenotype correlations have been reported. NF1 whole gene deletion is associated with facial dysmorphism and intellectual disability; missense variants affecting codon 844–848 were more prevalent in PN, symptomatic spinal neurofibromas, optic pathway gliomas, and skeletal abnormalities; c.2970_2972delAAT is a milder phenotype without neurofibromas; and missense variants affecting Met1149, Arg1276, Lys1423, and Arg1809 are associated with milder phenotype with Noonan-like features [36–41]. Moreover, these specific genotype–phenotype correlations and the impact of mutation types on phenotypes were also reported. Our recent study reported that NF1^+^ was more prevalent and the number of items included in NF1^+^ was higher in patients with truncating/splicing mutations and large deletions [17].

In the current study, the association between radiological findings and the NF1 functional domain affected by mutations were newly revealed in young pediatric patients because of high FASI frequency and radiologic progression which included newly developed optic pathway glioma, PN, and tumors. Neurofibromin modulates cell proliferation and differentiation by regulating the RAS signaling pathway. GRD is a catalytic domain of neurofibromin, which negatively regulates the RAS–MAPK signaling pathway by promoting the hydrolysis of the active form of RAS-GTP to an inactive form of RAS-GDP [6, 42]. Haploinsufficiency (*Nf1*^+/−^) or loss (*Nf1*^−/−^) of neurofibromin expression in neural stem cells showed growth and survival advantage with abnormal astroglial cell differentiation, which was rescued by the GRD expression [43].

A limitation of this study is that some clinical data were not evaluated or missed due to its retrospective nature. In particular, development assessment at initial evaluation was done by parents’ reports, which would have affected the relatively low prevalence of developmental delay in the patient cohort. Nevertheless, the findings of this study will improve the perception of the manifestations and genotype–phenotype correlation of NF1 in young children considering that WBMRI was performed in all participants and reviewed by an experienced pediatric radiologist.

## Conclusions

In conclusion, the current study provided some novel and important insights into the clinical and radiological manifestations in young pediatric NF1 patients evaluated by serial WBMRI and positive family history and genetic factors. In addition, the functional domain affected by *NF1* mutation (GRD domain in particular) contributes to the development of severe phenotype or disease progression. Future studies with long-term follow-up analysis in a larger patient cohort will provide a further understanding of its natural course and clinical and genetic contributing factors in young NF1 patients.

## Supplementary Information

**Additional file 1: Table S1**. Detailed imaging parameters of WBMRI. **Table S2**. The frequency of phenotypes according to the affected functional domains. **Table S3**. The detailed clinical and radiological findings of the patients who progress to NF1^+^.

**Additional file 2: Fig. S1**. A representative case of a 2-year-old boy with genetically confirmed NF type I (*NF1* c.539T > G (p.L180*)) showed radiologic progression during follow-up. **A**–**C** Images from baseline were WBMRI obtained at 2.91 years. **A**, **B** Coronal T2-weighted images with fat suppression show mild thickening with increased signal intensity in intraorbital segments of the bilateral optic nerves (arrows). **C** Coronal short tau inversion recovery (STIR) image demonstrates small high-signal nodular lesions at the small bowel mesentery, which suggests plexiform neurofibromas. **D**–**F** Images from follow-up WBMRI were obtained at age of 4.75 years. **D** Progression of thickening with increased signal intensity involving both optic nerves was noted on coronal T2WI with fat suppression (arrows). **E** Coronal STIR image shows markedly increased size of the plexiform neurofibromas involving small bowel mesentery (arrows). Newly developed plexiform neurofibromas were noted in the right-sided pelvic cavity (thick arrow) and dome of the urinary bladder (broken arrow). **F** Another new plexiform neurofibromas are seen at the right 12th intercostal space (arrows).

## Data Availability

Anonymized data not included in this article will be shared by request from any qualified investigator.

## Abbreviations

CALMs: Café-au lait macules
CSRD: cysteine-serine rich domain
FASI: Focal areas of signal intensity
GRD: GTPase-activating protein-related domain
KICDT: Korean infant and child development test
MPNST: Malignant peripheral nerve sheath tumor
NF1: Neurofibromatosis type 1
NIH: National Institutes of Health
WBMRI: Whole body magnetic resonance imaging
