# Supplementary figures and images for: Whole-body MRI evaluation in neurofibromatosis type 1 patients younger than 3 years old and the genetic contribution to disease progression
Source: Orphanet J Rare Dis. 2022 Jan 29;17:24. doi: 10.1186/s13023-022-02174-3 (PMC8800361; doi:10.1186/s13023-022-02174-3)

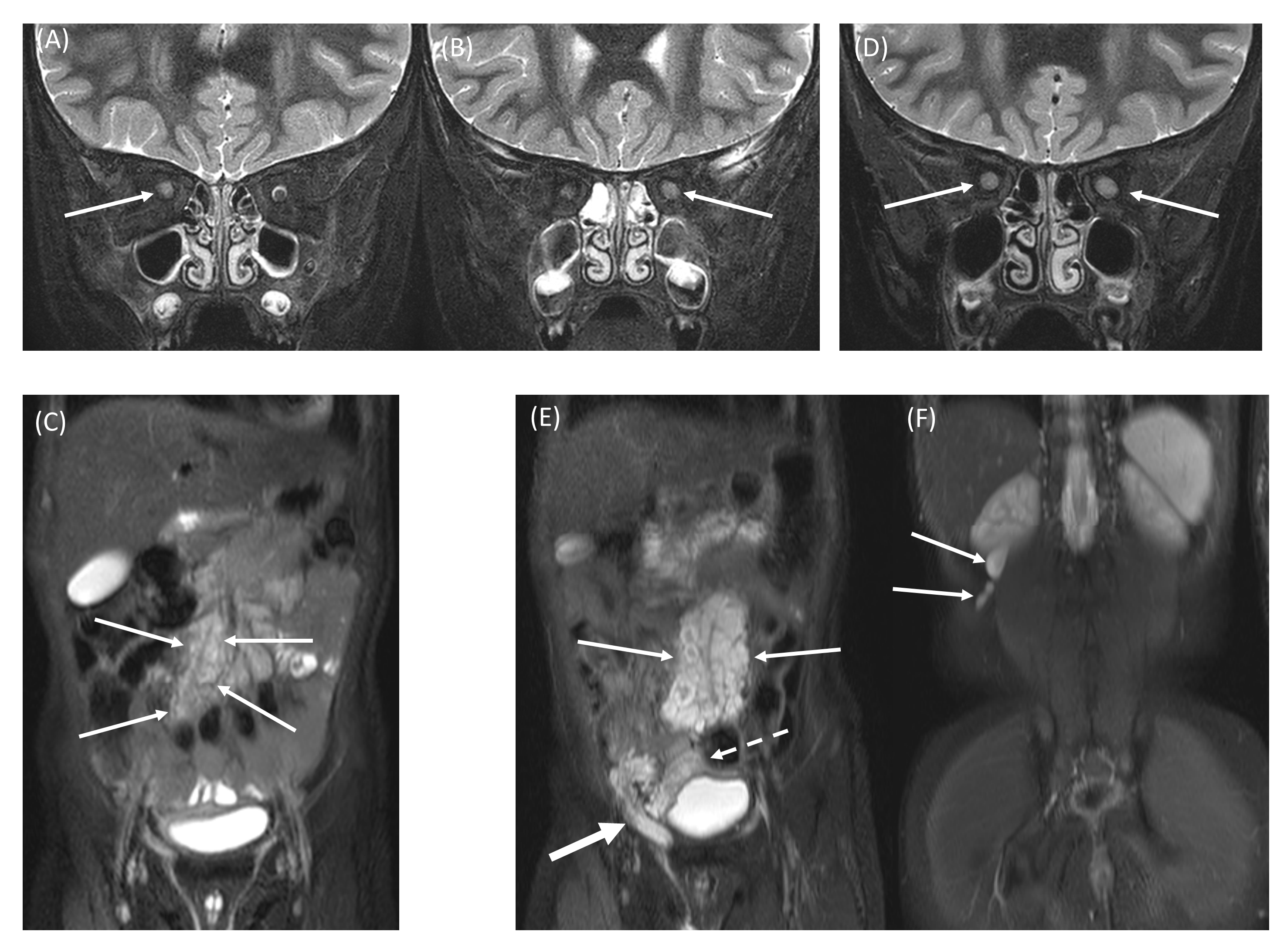

Supplement: Supplementary file 2 — Additional file 2: Fig. S1. A representative case of a 2-year-old boy with genetically confirmed NF type I (NF1 c.539T > G (p.L180*)) showed radiologic progression during follow-up. A–C Images from baseline were WBMRI obtained at 2.91 years. A, B Coronal T2-weighted images with fat suppression show mild thickening with increased signal intensity in intraorbital segments of the bilateral optic nerves (arrows). C Coronal short tau inversion recovery (STIR) image demonstrates small high-signal nodular lesions at the small bowel mesentery, which suggests plexiform neurofibromas. D–F Images from follow-up WBMRI were obtained at age of 4.75 years. D Progression of thickening with increased signal intensity involving both optic nerves was noted on coronal T2WI with fat suppression (arrows). E Coronal STIR image shows markedly increased size of the plexiform neurofibromas involving small bowel mesentery (arrows). Newly developed plexiform neurofibromas were noted in the right-sided pelvic cavity (thick arrow) and dome of the urinary bladder (broken arrow). F Another new plexiform neurofibromas are seen at the right 12th intercostal space (arrows). [file 13023_2022_2174_MOESM2_ESM.tif]
